# Supplementary material for: Profitability determinants of the natural stone industry: Evidence from Spain and Italy
Source: PLoS One. 2022 Dec 7;17(12):e0276885. doi: 10.1371/journal.pone.0276885 (PMC9728913; doi:10.1371/journal.pone.0276885)
Supplement: Attachment — Submitted filename: Review Results_XX.docx [file pone.0276885.s001.docx]

1. ABSTRACT — at the end of the passage, add the implication of your research, how it might be beneficial for company and government especially in the country where the research is conducted.
2. INTRODUCTION — need to be made more interesting, structured like an upside-down pyramid: (1) start from the general view of the research and why the object is intriguing, especially in those two countries, (2) theories surrounding the profitability, (3) existing research about profitability and how your research is filling the gap in the literature, (4) closed with the research aim and contribution.

The first paragraph also doesn’t seem to be needed, instead start with what’s interesting about the research object.

3. LITERATURE REVIEW—this section is also not structured in a good way for readers to be able to comprehend the red string of the research. This section must be filled with the theories that theories used as a basis in the research and description of all the variables, also older studies that support your hypothesis. That way conceptual framework can be built and understood clearly.
4. METHODOLOGY
a) Sample selection & data collection: elaborate more on the sampling technique and how you gathered 453 samples, and why the period 2015-2019 was selected. It’s good to be more update since this is already 2022.
b) Description of variables: in line 174 and 175, there are incorrect citing.
5. RESULTS — beside presenting the result of descriptive statistic, also tell systemically how your hypothesis is proven or not proven in each country. It is meant to answer the research question.
6. DISCUSSION — must include a discussion of every result you presented in previous section. Explain the implications of the research result for firms and countries. Lines 435-450 are not necessary in this section because it has been explained before. Focus to the discussion of result in each country. To make it more interesting, explain the contrast between those two countries and what’s your analysis regarding the difference.
7. CONCLUSION — at the end of the conclusion, add the limitation of this research and the direction of future research that you suggest.
